# Baicalin reduces sunitinib-induced cardiotoxicity in renal carcinoma PDX model by inhibiting myocardial injury, apoptosis and fibrosis

**DOI:** 10.3389/fphar.2025.1563194

**Published:** 2025-04-08

**Authors:** Zefu Yang, Jianping Wan, Xinjin Zhang, Jiaqi Mei, Hua Hao, Sibo Liu, Yun Yi, Meixiu Jiang, Yuanqiao He

**Affiliations:** ^1^ Cardiovascular Medicine Department of Nanhai District People’s Hospital, Foshan, Guangdong, China; ^2^ Cardiovascular Medicine Department of The Sixth Affiliated Hospital, School of Medicine, South China University of Technology, Foshan, China; ^3^ Electrophysiology Department of The Sixth Affiliated Hospital, School of Medicine, South China University of Technology, Foshan, China; ^4^ Cardiovascular Center, Affiliated Hospital of Yunnan University, Kunming, China; ^5^ Department of Hematology, The Second Affiliated Hospital, Jiangxi Medical College, Nanchang University, Nanchang, Jiangxi, China; ^6^ Department of Pathology, Yangpu District Central Hospital, School of Medicine, Tongji University, Shanghai, China; ^7^ The Queen MARY school, Jiangxi Medical College, Nanchang University, Nanchang, China; ^8^ Center of Biobank, Nanchang University Second Affiliated Hospital, Jiangxi Medical College, Nanchang, China; ^9^ The Institute of Translational Medicine, Jiangxi Medical College, Nanchang University, Nanchang, China; ^10^ Center of Laboratory Animal Science, Nanchang University, Nanchang, China; ^11^ Jiangxi Province Key Laboratory of Laboratory Animal, Nanchang, China; ^12^ Nanchang Royo Biotechnology, Nanchang, China

**Keywords:** baicalin, sunitinib, cardiotoxicity, renal carcinoma, PDX

## Abstract

Sunitinib (SU), a multi-targeted tyrosine kinase inhibitor, has anticancer function but its clinical use is often limited by cardiovascular complications. Baicalin (BA) has demonstrated various pharmacological activities including antioxidant, anti-inflammatory and antiviral properties, but its potential roles in SU-induced cardiotoxicity have not been reported. In this study, we aimed to investigate the effect of BA in SU-induced cardiotoxicity *in vivo* by using renal carcinoma patient-derived xenograft (PDX) model. Female Nod Scid mice with renal carcinoma PDX were treated with vehicle, SU (50 mg/kg/d), BA (100 mg/kg/d), or BA combined with SU for 6 weeks. The tumor volume and weight of tumor-bearing mice were measured, and cardiovascular functions were evaluated by testing the Heart index and blood biochemical indicators, and by hematoxylin and eosin (H&E), Masson and Tunel staining. The results showed that SU therapy and combination therapy effectively inhibited the growth of renal tumors. Combination therapy inhibited SU-induced increase of creatine kinase (CK) and lactate dehydrogenase (LDH), and ameliorated the heart parameters. Moreover, BA effectively protected SU-induced cardiac dysfunction by decreasing injury, apoptosis, and fibrosis. Collectively, our results demonstrate that BA can be as a potential cardioprotective approach for cardiovascular complications during SU regimen.

## Introduction

Renal cell carcinoma (RCC) refers to the cancer originating from renal epithelium, accounting for more than 90% of renal cancer (Hsieh et al., 2017). Up to 30% of patients with RCC have metastatic diseases. However, traditional chemotherapy and cytokine therapy may not be tolerated for this kind of patients and bring greater adverse reactions (Motzer et al., 2000; Motzer et al., 2007). The introduction of targeted therapy based on Sunitinib (SU) has greatly improved the anti-tumor efficacy and fewer toxic side effects, which is a breakthrough in the treatment of metastatic RCC (Motzer et al., 2007).

SU is a novel oral multi-target tyrosine kinase inhibitor with anti-tumor and anti-angiogenesis activities (Le Tourneau et al., 2007). Since the launch of a variety of targeted drugs in 2006, SU has become the most commonly used first-line treatment for metastatic RCC (Goodman et al., 2007). However, such antiangiogenic drugs have been proven to cause cardiovascular toxicity in clinical use (Di Lorenzo et al., 2009; Goodman et al., 2007). For example, it is reported that hypertension or congestive heart failure are the most common side effects in patients taking SU during treatment (Gupta and Maitland, 2011). In addition, SU has been reported to cause left ventricular dysfunction more than other kinase inhibitors (KI), which may be related to its inhibition of PDGFR pathway (Chintalgattu et al., 2010). It was not completely reversible in most patients, even after termination with SU, which limited its clinical application (Chu et al., 2007; Gan et al., 2009; Khakoo et al., 2008; Telli et al., 2008; Xu et al., 2022). This calls for developing strategies for the prevention and treatment of SU-induced cardiotoxicity that specifically act cardiovascular-wise and do not compromise its tumor-killing potency.

Baicalin (BA), the main active ingredient of the widely used Chinese herbal medicine scutellaria baicalensis, has antioxidant, anti-inflammatory and antiviral properties (Luan et al., 2015). Studies indicate that baicalin exhibits some anti-tumor properties. Baicalin can exert antitumor and apoptosis-inducing effects by targeting Annexin A2 (Kusakabe et al., 2024). Baicalin also reduces chronic stress-induced breast cancer metastasis via directly targeting β2-adrenergic receptor (Jia et al., 2024), and induces cell death of non-small cell lung cancer cells via MCOLN3-mediated lysosomal dysfunction and autophagy blockage (Dong et al., 2024). Moreover, it is an effective angiogenesis inhibitor, which has been proven to inhibit the proliferation, migration, and differentiation of endothelial cells (Liu et al., 2003). Recent studies have shown that BA has a protective effect on myocardium (Luan et al., 2019), and is effective in animal models of various cardiovascular diseases, such as pulmonary hypertension, atherosclerosis and myocardial ischemia (Liu et al., 2014). Therefore, we speculate that BA has a protective effect on SU-induced cardiotoxicity.

In this study, we aimed to investigate the role of BA on SU-induced cardiotoxicity by using the RCC-PDX model. Our results demonstrated that BA effectively protected SU-induced cardiac dysfunction by decreasing injury, apoptosis, and fibrosis. Collectively, our results suggest that BA can be as a potential cardioprotective approach for cardiovascular complications during SU regimen.

## Materials and methods

### Regents

BA and SU were obtained from Selleckchem (Houston, America), and both were dissolved in DMSO and diluted with 0.9% NaCl.

### Animals

All animal-related experimental procedures and methodologies were complied with the Guide for the Care and Use of Laboratory Animals published by NIH and approved by the Animal Ethics Committee of Nanchang Royo Biotech company. The approval date is 1 June 2022. Institutional Animal Care and Use Committee Number is 2,022,030,601.

Eight-week-old Nod Scid female mice were from Hangzhou Ziyuan Laboratory Animal Technology Co., and were maintained under standard temperature (23°C ± 3°C) and lighting conditions (12 h dark/12 h light cycles). Approximately 1 week was provided before starting experiments to allow the animals to acclimate to the laboratory environment.

### PDX model construction

All human-related experimental procedures and methodologies were approved by the medical ethics committee of the Second Affiliated Hospital of Nanchang University. Written informed consent was obtained from the individual (s) before sample collection. The approval date is 5 August 2021. The medical ethics committee number is Permit No. 2021012.

Fresh renal carcinoma material was obtained from a 53-year-old male patient from the second affiliated hospital of Nanchang University. The fresh renal carcinoma tissue from the patient was cut into small fragments of 2 mm × 2 mm × 2 mm, and then the tissue fragments were subcutaneously inoculated into the scapula of Nod Scid mice. When the volume of PDX mice tumor increased to more than 1,000 mm^3^, it was removed and inoculated into other mice as same as the parental tumor. In this way, when the tumor was transmitted to the third generation (P3), the next-generation of tumors were used for experiments. Tumor volumes were calculated as (length × width^2^)/2.

### The *in Vivo* study

When the tumors reached a mean size of 50 mm^3^, the mice were randomly divided into four groups (n = 8), and accepted the following treatment: Group 1 (Control group); Group 2 (Sunitinib), the mice were treated with 50 mg/kg/d SU through intragastric administration; Group 3 (baicalin), the mice were treated with 100 mg/kg/d BA through intraperitoneal injection; Group 4 (sunitinib combined with baicalin), the mice were treated with 50 mg/kg/d SU through intragastric administration and 100 mg/kg/d BA through intraperitoneal injection. The concentrations of SU and BA used for the *in vivo* experiments were established based on results from earlier studies (Sourdon et al., 2021; Liu et al., 2014). All the mice were treated for 6 weeks. The body weight and tumor volumes were measured every 3 days. At the end of the experiments, all of the mice were euthanized by CO_2_ followed by collection of mouse blood, heart, and tumor. The blood was harvested for analysis of involved biochemical metrics, the hearts were harvested for weight measurement, photograph and staining, and the tumors were harvested for weight measurement and photograph.

### Efficacy evaluation

The effect of the drug is evaluated by tumor proliferation rate (T/C) and tumor growth inhibition (TGI). T/C was calculated as the individual relative tumor volume (RTV) of treatment group compared to the control group. RTV was calculated as V_t_/V_0,_ in which Vt is the tumor volume at each measurement and V0 is the initial tumor volume. TGI was calculated as (1-T/C) x 100%, in which T is the average tumor weight of the treatment group and C is the average tumor weight of the control group. The effect of the drug is considered as valid when T/C < 60% or TGI ≥30%.

## PCR

RNA was extracted on ice from PDX tumor tissues. Primer premier 5.0 design software was used to design human -derived genome-specific primers for GAPDH (hs-F1: GGC​TCT​TAA​AAA​GTG​CAG​GGT​C; Hs-R1: ATG​GTA​CAT​GAC​AAG​GTG​CGG; hs-F2: TAA​CTG​TCT​GCT​TCT​CTG​CTG​TAG​GC; Hs-R2: GCT​TCA​CCA​CCT​TCT​TGA​TGT​CAT​CA) and mouse-derived genome-specific primers for GAPDH (mus-F1: CAG​GTT​GTC​TCC​TGC​GAC​TT; mus-R1: CAG​CTG​GAT​GTC​AGA​GCC​AA; mus-F2: AAG​GGC​ATC​TTG​GGC​TAC​AC; mus-R2: CCT​GCT​TCA​CCT​CCC​CAT​AC). The extracted sample was amplified by PCR as described previously (Jiang et al., 2024; Zeng et al., 2023). Five microliters DNA Marker (DL 2000) was added as a reference for the length of the amplified fragment. Electrophoresis was performed at 100 V voltage for 15 min. When the indicator bromophenol moved to 2/3 of the gel, electrophoresis was terminated. The PCR Product was observed in the DNA gel electrophoresis imager and photographed for analysis.

### Histopathological staining

Heart samples were fixed in 4% paraformaldehyde/PBS, embedded in paraffin, and sectioned at 5 µm intervals. After de-waxing and rehydration, the sections were stained with H&E, Tunel staining, and Masson’s trichrome. All images were captured with an Olympus microscope. H&E staining was conducted to evaluate the severity of cardiac damage by observing the cardiac cells and the nucleus of myocardial fiber cells. The damage quantification was determined as reported (AlAsmari et al., 2022). In briefly, the myocardial tissue was graded using the following parameters: nuclear enlargement and inflammation based on a four-score evaluation system (0, histopathological changes = 1–25%; 1, histopathological changes = 26–50%; 2, histopathological changes = 51–75%; and 3, histopathological changes = 76–100%). This procedure was conducted in at least 10 random areas in each heart section, in three animals from each group. The mean score for each parameter was calculated and subjected to statistical analysis.

### Tunel staining

Mouse heart cryosections were stained using CF488 Tunel Cell Apoptosis Detection Kit (G1504, Servicebio, China) according to the manufacturer’s protocols, and counterstained with DAPI to visualize nuclei. The images were photographed using confocal laser scanning microscopy (Zeiss, Oberkochen, Germany). The apoptosis quantification was performed by using mean fluorescence intensity (MFI) in six animals from each group.

### Masson staining

Paraffin sections were dewaxed, washed, and dyed with Regaud’s hematoxylin for 5–10 min and Masson Ponceau S acid fuchsin stain for 5–10 min. Subsequently, sections were washed in 2% aqueous glacial acetic acid and then differentiated in 1% aqueous phosphomolybdic acid for 3–5 min. Afterwards, sections were stained with aniline blue for 5min and washed. The slices were dehydrated and finally sealed with neutral gum. Collagen volume fraction (CVF) = collagen area/total area.

### Biochemical analysis

Blood was kept for about 1 h at room temperature (RT). After centrifugation for 10 min at 4,000 rpm, the serum was transferred into a new test tube and kept at −80°C. The level of serum TP (Total Protein), AST (Aspartate Transaminase), ALT (Alanine Transferase), ALP (alkaline phosphatase), LDH, CK, Crea (Creatinine), BUN (Blood urea nitrogen), and GLU (Glucose) were determined using commercially Chemistry Reagent Disc purchased from Seamay (Chengdu, China) to examine the function of heart, liver and kidney.

### Statistics analysis

All data are expressed as the mean ± standard error of the mean (SEM) with GraphPad Prism software 7.0. For statistical data of group design, statistical analysis between groups was performed by t-test if the normal distribution and homogeneity of variance were satisfied, otherwise non-parametric test was used. For the single-factor design with multiple levels, ANOVA was used if the normal distribution and homogeneity of variance were satisfied. Non-parametric test Kruskal–Wallis testing and SNK testing were used to compare the differences between two groups, otherwise. Differences p < 0.05 were considered statistically significant.

## Results

### Renal carcinoma PDX model was constructed successfully

**FIGURE 1:**
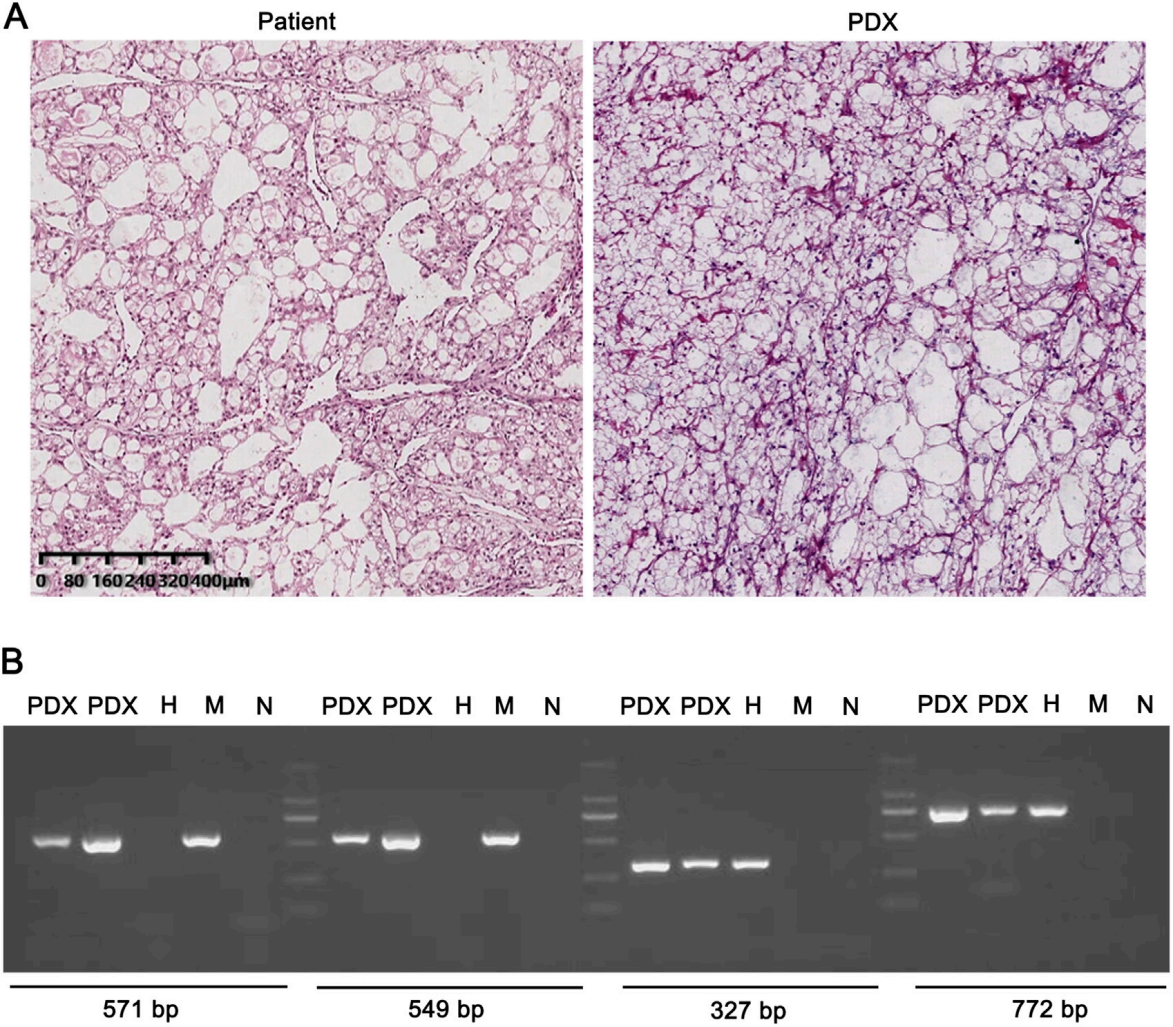
The PDX tissue is consistent with the patient’s tissue **(A)** Tissues from PDX model and patient cancer were consistent in morphological characteristics **(B)** The PDX tissue samples contained both mouse and human target genes GAPDH. H: human; M: mouse; N: negative.

We successfully constructed a PDX model of renal carcinoma. The morphological characteristics of tumor tissue and PDX model were observed by H&E staining. Both PDX and patient-derived tissues showed typical kidney renal clear cell carcinoma tissue characteristics, which represented that the cancer cells were arranged in nests and sheets, the cytoplasm of cells was bright, and the chromatin was fine-grained (Figure 1A). Tissues from PDX model and patient cancer were consistent in morphological characteristics. We also determined if renal carcinoma PDX tissue was human genes by PCR. The PDX tissue samples contained both mouse and human target genes, which ruled out spontaneous tumors of mice (Figure 1B). The renal carcinoma PDX is one human origin cancer model.

### SU and SU plus BA inhibited tumor growth in renal carcinoma PDX model

**FIGURE 2:**
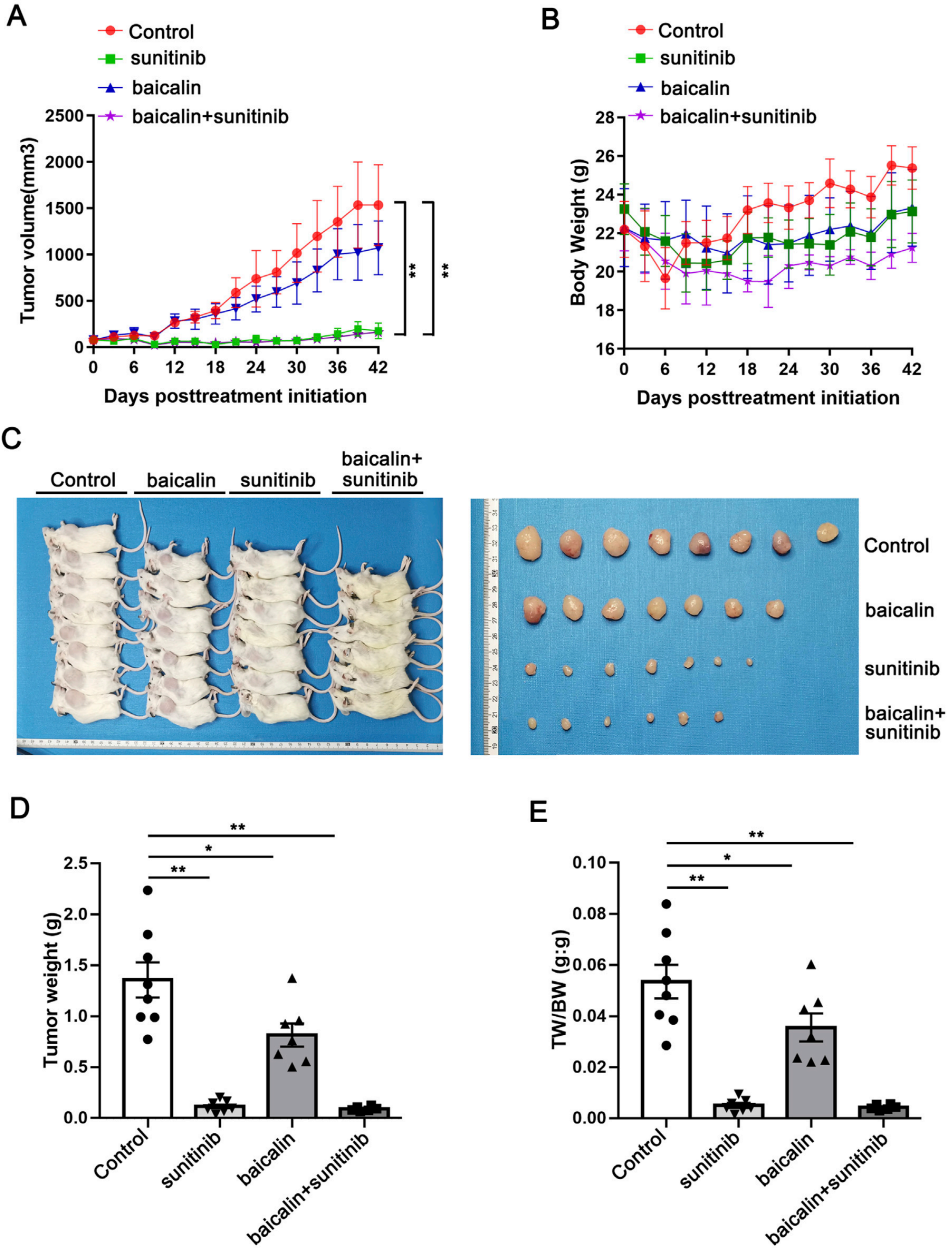
Sunitinib and sunitinib co-treatment with baicalin inhibited the growth of tumor **(A)** The tumor volume of Control, sunitinib, baicalin, and sunitinib-baicalin-treated mice (n = 6–8) **(B)** The body weight of Control, sunitinib, baicalin, and sunitinib-baicalin-treated mice (n = 6–8) **(C)** General and tumor photograph of Control, sunitinib, baicalin, and sunitinib-baicalin-treated mice (n = 6 or 8) **(D)** The tumor weight of Control, sunitinib, baicalin, and sunitinib-baicalin-treated mice (n = 6–8) **(E)** The ratio of tumor weight and body weight (TW:BW) of Control, sunitinib, baicalin, and sunitinib-baicalin-treated mice (n = 6–8). *P < 0.05, **P < 0.01.

Firstly, we examined the effect of SU and BA on the growth of tumor. The mice were treated with the vehicle, SU, BA, or SU and BA for 42 days, and the tumor volume and weight were monitored every 3–4 days. As shown in Figure 2A, consistent with the reports from the clinic and investigative, SU markedly inhibited the growth of tumor. And co-treatment of BA and SU also significantly inhibited the growth of tumor. However, they had little effect on the body weight (BW) of mice (Figure 2B). Moreover, at the end of the experiments, we isolated the tumor, measured the weight and photographed it. As shown in Figure 2C, SU, BA, and co-treatment significantly reduced the size of tumor. Accordingly, SU, BA and the co-treatment of SU and BA significantly decreased tumor weight (Figure 2D). Similarly, SU, BA, and BA combined with SU significantly reduced the ratio of tumor weight and body weight (Figure 2E).

**FIGURE 3:**
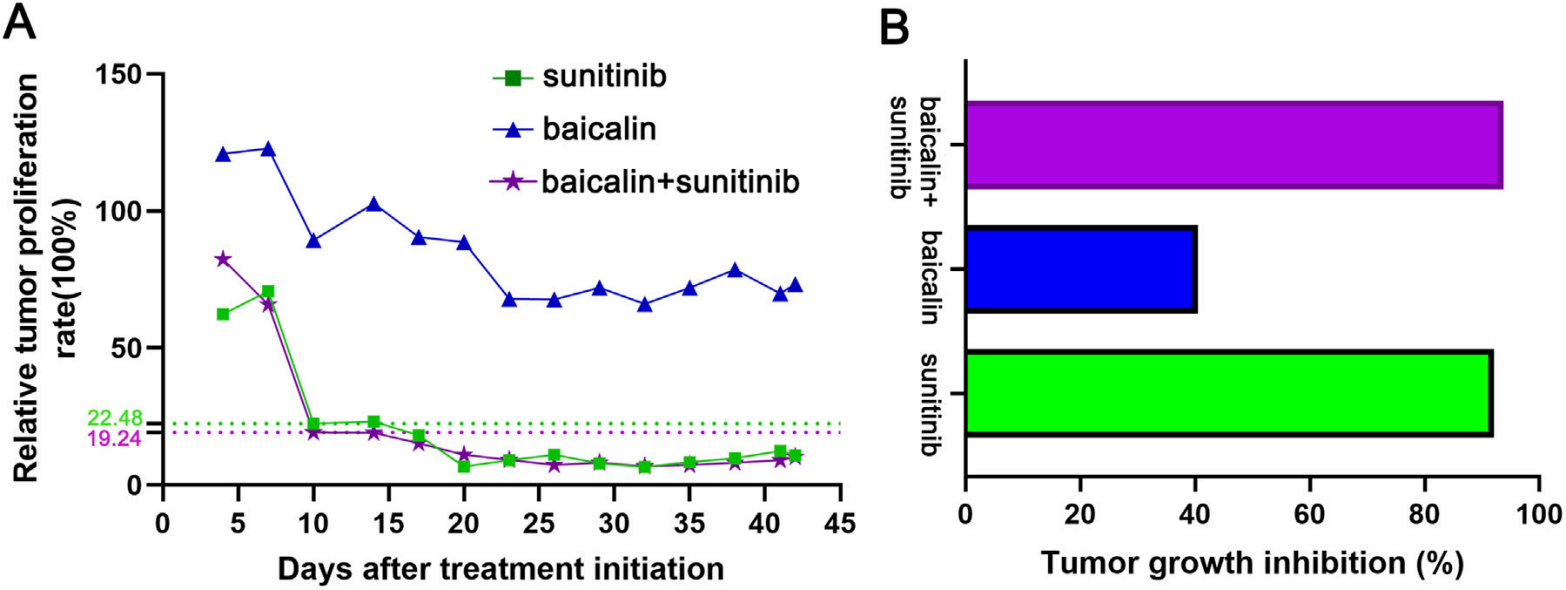
T/C and TGI were calculated to validate the effectiveness of the drug **(A)** T/C (tumor proliferation rate) was calculated as the RTV of sunitinib, baicalin, and sunitinib-baicalin-treated group compared to the Control group. The effect of the drug is considered as valid when T/C<60% **(B)** TGI (tumor growth inhibition) was calculated as (1-T/C) x 100%, in which T is the average tumor weight of the treatment group and C is the average tumor weight of the control group. The effect of the drug is considered as valid when TGI ≥30%.

In order to further validate the effectiveness of the drug, we calculated the T/C and TGI. As shown in Figure 3A, SU and the combination of SU and BA highly decreased the T/C on day 10 (22.48 and 19.24), but BA slightly inhibited the T/C (66.06). Moreover, the TGI in SU and the co-treatment group is much greater than 30% (91.54% and 93.25% respectively), while the TGI of BA is slightly greater than 30% (39.95%) (Figure 3B). Taken together, all these results indicate that SU and the co-treatment highly inhibited tumor growth.

### BA reduced SU-induced cardiotoxicity

**FIGURE 4:**
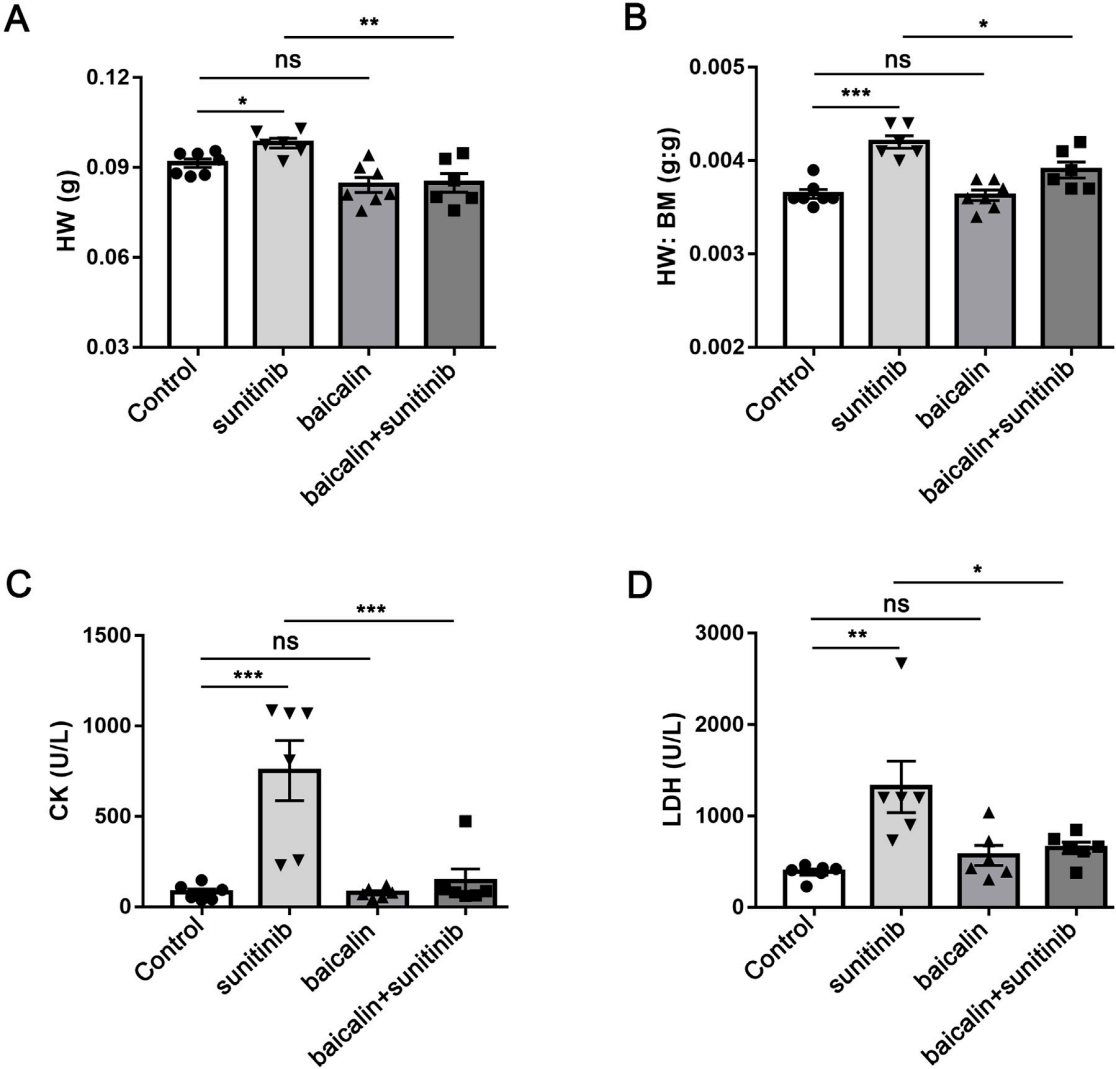
Baicalin reduced sunitinib-induced cardiotoxicity **(A)** The heart weight of Control, sunitinib, baicalin, and sunitinib-baicalin-treated mice (n = 6–8) **(B)** The ratio of heat weight and body weight was calculated in Control, sunitinib, baicalin, and sunitinib-baicalin-treated mice (n = 6–8) **(C)** The serum CK level of Control, sunitinib, baicalin, and sunitinib-baicalin-treated mice **(D)** The serum LDH level of Control, sunitinib, baicalin, and sunitinib-baicalin-treated mice. *P < 0.05, **P < 0.01; ***P < 0.001.

To evaluate the effects of BA on SU-induced cardiotoxicity, we examined the cardiovascular parameters of the treated mice. Although SU-treated mice did not significantly exhibit elevated the heart weight (HW) compared with vehicle-treated mice, co-treatment of BA and SU markedly decreased HW of mice (Figure 4A). However, SU-treated mice showed highly elevated HW/BW, and BA combined with SU reversed the effects (Figure 4B). Furthermore, the increased serum level of CK and LDH by SU was significantly alleviated by BA co-treatment with SU (Figures 4C, D). However, the other blood biochemical indexes of SU treated mice had no significant difference compared with those of the control (Table 1). These results demonstrate that BA co-treatment with SU ameliorates SU-induced cardiotoxicity.

**TABLE 1:** The influence of different antineoplastic drugs on blood biochemical indexes (x ® + s).

Group	TP (g/L)	AST (U/L)	ALT (U/L)	ALP (U/L)	Crea (umol/L)	Urea (umol/L)	GLU (mmol/L)
Control	52.67 ± 5.37	88.57 ± 52.7	41.29 ± 34.06	79.14 ± 13.88	19.42 ± 2.06	8.06 ± 0.97	11.81 ± 1.85
Sunitinib	60.37 ± 10.45	336.71 ± 326.89	92 ± 61.78	114.29 ± 32.67	13.95 ± 3.37	8.7 ± 2.41	7.46 ± 0.72
Baicalin	58.96 ± 4.31	167.86 ± 150.48	92.43 ± 87.81	83 ± 18.26	14.7 ± 2.83	9.1 ± 0.69	10.52 ± 1.47
Sunitinib + Baicalin	55.23 ± 4.66	133.17 ± 31.21	46.33 ± 13.95	93.67 ± 14.24	18.94 ± 6.52	6.91 ± 0.83	9.63 ± 0.82

### BA inhibited SU-induced myocardial injury, apoptosis, and fibrosis

**FIGURE 5:**
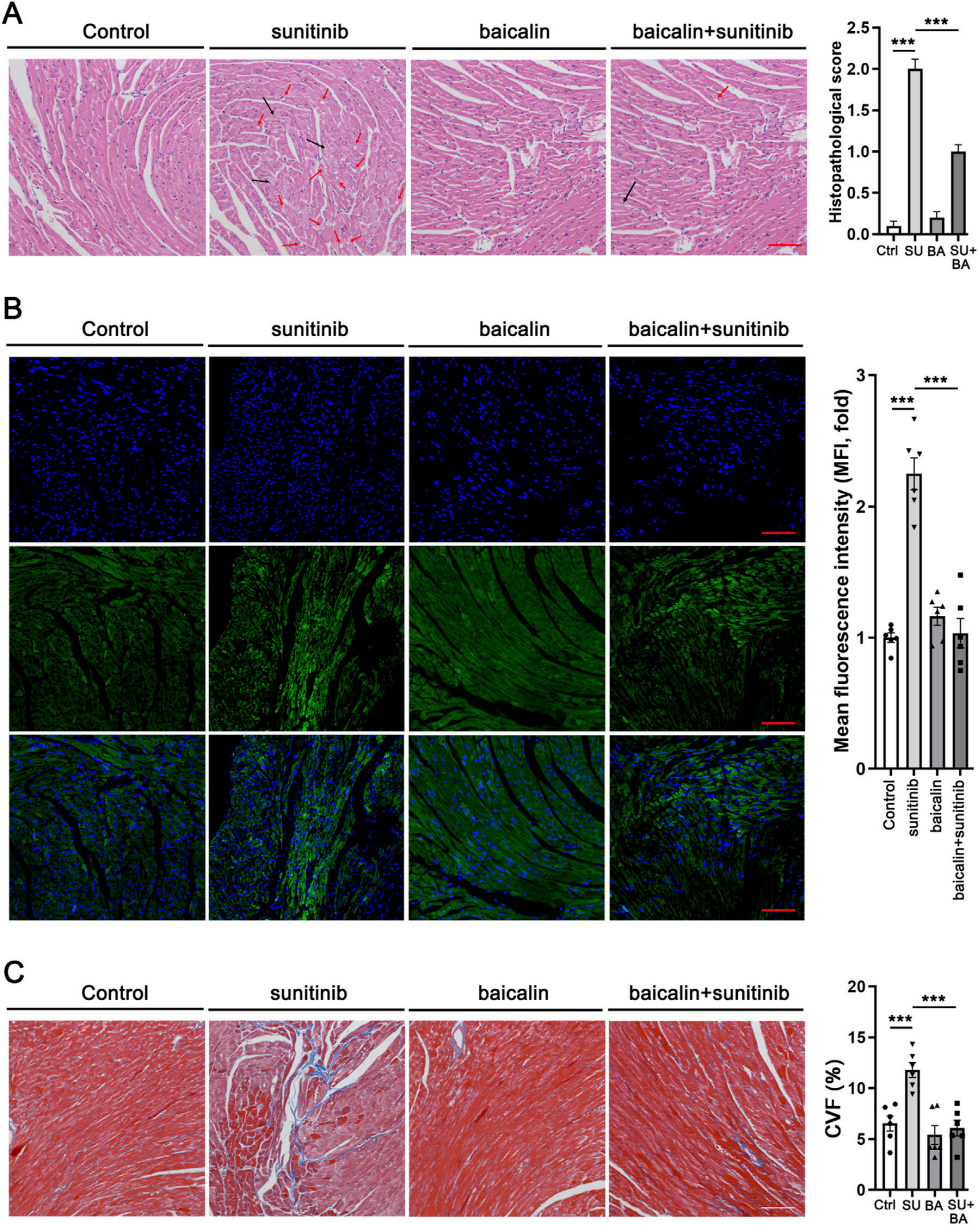
Baicalin inhibited sunitinib-induced myocardial injury, apoptosis and fibrosis **(A)** The H&E staining of Control, sunitinib, baicalin, sunitinib-baicalin-treated mice and Histopathological score. The red arrows represent cytoplasmic vacuolization; the black arrows represent tissue edema and structural disorders **(B)** The Tunel staining of Control, sunitinib, baicalin, sunitinib-baicalin-treated mice and quantification **(C)** The Masson staining of Control, sunitinib, baicalin, sunitinib-baicalin-treated mice and quantification. Scale bar, 100 µm.

We then sought to explore the mechanisms underlying these changes. Histological analysis of SU-treated mouse hearts by H&E staining identified the loss of cytoplasm (cytoplasmic vacuolization) and disorganization of the myofibrils, suggesting the existence of cardiomyocyte death, which was defined as myocardial injury. However, co-treatment of BA and SU reversed these effects (Figure 5A). Because apoptosis is the most common cause of cell death, we further analyzed the cardiomyocyte apoptosis in the heart of the treated mouse. Tunel staining showed that BA co-treatment with SU ameliorated SU-induced apoptosis of cardiomyocytes in the heart (Figure 5B). Masson staining indicated that BA co-treatment with SU ameliorated SU-induced fibrosis of cardiomyocytes in the heart (Figure 5C). Taken together, BA inhibited SU-induced myocardial injury, apoptosis and fibrosis.

## Discussion

**FIGURE 6:**
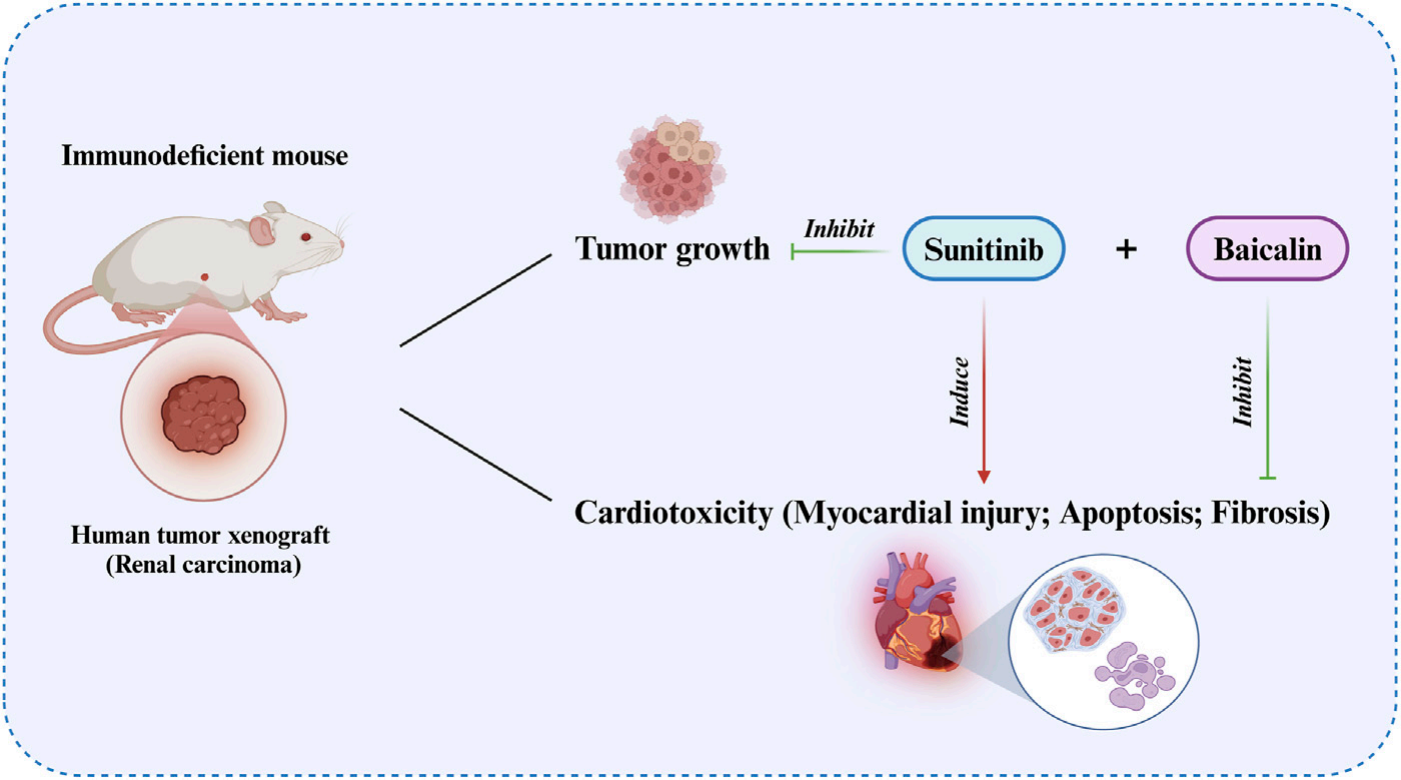
Proposed model of baicalin inhibiting sunitinib-induced cardiotoxicity Baicalin could alleviate sunitinib-induced cardiotoxicity by decreasing the sunitinib-increased cardiomyocyte injury, apoptosis, and fibrosis.

SU has shown significant clinical benefits in the treatment of advanced or metastatic RCC. However, cardiovascular toxicity following SU treatment is the major obstacle for its wide safe use in clinic. Developing prevention and treatment strategies for SU-induced cardiotoxicity is urgent to improve survival of RCC patients. BA has been reported to be cardioprotective in a range of cardiovascular conditions, but its effects in SU-induced cardiotoxicity remain unknown. In our present study, we used the PDX models to find that BA could alleviate SU-induced cardiotoxicity by decreasing the SU-increased apoptosis and fibrosis of cardiomyocytes in the heart (Figure 6). These results may provide potential therapeutic strategies for SU-induced cardiotoxicity.

PDX models have garnered increasing attention for cancer research since the last decade (Zhuo et al., 2020). These models are typically characterized by the implantation of fresh patient-derived tumor tissues into immunodeficient mice. Currently, cell-line xenografts are the standard for preclinical research (Bhimani et al., 2020). However, they do not accurately reflect the true behavior of the host tumor and are able to adapt to *in vitro* growth, losing the original properties of the host tumor (Bhimani et al., 2020; Hidalgo et al., 2014). Trials examining PDX models have shown that they mostly retain the principal histological and genetic characteristics of their donor tumor and remain stable across passages (Abdolahi et al., 2022; Bhimani et al., 2020; Hidalgo et al., 2014; Tentler et al., 2012). They are able to accurately replicate tumor growth, diversity of tumor cells, and tumor progression, including metastatic potential (Bhimani et al., 2020; DeRose et al., 2011; Hidalgo et al., 2014; Tentler et al., 2012). Therefore, these models have been successfully used to be predictive of clinical outcomes and for preclinical drug evaluation, biomarker identification, biological studies, and personalized medicine strategies (Evrard et al., 2020; Hidalgo et al., 2011; Hidalgo et al., 2014). In our study, we used the tumor tissues from the patient with renal carcinoma to establish the PDX models and examine the effect of BA on SU-induced cardiotoxicity. Our study effectively mimics the state of patients taking SU in the clinic, and may better screen effective drugs that treat SU-induced cardiotoxicity.

SU, defined as a selective tyrosine kinase inhibitor, plays an important role in anti-cancer especially in RCC. However, several studies have shown that cardiovascular adverse effects of SU commonly include hypertension, decreased LVEF (Left Ventricular Ejection Fractions), and congestive heart failure (Chu et al., 2007; Kimura et al., 2017; Lavandero et al., 2015; Yang et al., 2019). The myocardial injury caused by cardiotoxic drugs promotes compensative remodeling, manifested by myocardial fibrosis, hypertrophy and other morphological changes (Xu et al., 2022). Myocardial fibrosis is thought to be an early manifestation and the hallmark of hypertrophic cardiomyopathy (Ho et al., 2010). Here, we confirmed that treatment with SU markedly inhibited tumor growth, at the same time, led to increased HW/BW, serum level of CK and LDH, myocardial fibrosis, and apoptosis, compared to control group. Indeed, SU inhibited tumor growth and induced cardiotoxicity. BA has been proven to have anti-tumor effect in clinic and is effective for a variety of tumors, such as osteotropic breast cancer and acute lymphoblastic leukemia (ALL) (Orzechowska et al., 2020; Wang et al., 2020). Moreover, BA was reported to have some cardiovascular protective effects, therefore, we speculate that BA has a protective effect on SU-induced cardiotoxicity. In the present study, we found BA alone reduced the tumor weight to some extent, and combined with SU significantly inhibited tumor growth, decreased tumor weight, and reversed SU-induced cardiotoxicity by decreasing SU-increased cardiomyocyte injury, apoptosis and fibrosis.

However, the involved molecular mechanisms by which BA reduces SU-induced cardiotoxicity remain unknown. BA was reported to significantly reduce the infarct area and myocardial enzymes (CK, CK-MB, LDH, and cTnT) and inhibit the activity and protein expression of Caspase-3, which was related to the mitogen-activated protein kinases (MAPK) cascade in acute myocardial infarction in rats (Liu et al., 2013). Moreover, BA could inhibit the expression of fibrosis-related factors (MMP-9, MMP-2, CTGF, and TGF-β1) in ventricular myocytes and the activation of endoplasmic reticulum stress (ERS) in the heart (Dai et al., 2017; Shen et al., 2014). It has been also shown to reduce pressure overload-induced cardiac dysfunction and ventricular remodeling by inhibiting myocardial hypertrophy, fibrosis, apoptosis metabolic abnormalities and restoration of PPARβ/δ (Zhang et al., 2017). Although BA reduces ERS in cardiomyocytes as well as restores PPAR α and PPAR β/δ pathway to reverse myocardial injury and restore left ventricular remodeling, there was no significant difference in left ventricular hypertrophy between mice in the combination group and SU group in this experiment (Luther et al., 2012). Moreover, Li et al. revealed that enhanced PI3K activity protects cardiomyocytes from SU-induced calcium dysregulation and contractile dysfunction, which suppressed SU-induced cardiotoxicity both *in vitro* and *in vivo* (Liu et al., 2014). Therefore, in the present study, we speculate that BA may regulate MAPK or PPARα or PPAR β/δ or PI3K pathways to inhibit caspase-3, MMP, and TGF-β, thus decreased SU-induced cardiotoxicity. We will examine these signaling pathways to clarify the involved mechanisms in the future experiments. Moreover, there are still some limitations to our research. For example, firstly, the PDX model is both time-consuming and expensive; secondly, we did not perform the echocardiography or measure the blood pressure of the mice because of the pandemic. However, this did not affect the accuracy of our experimental conclusions.

## Conclusion

Collectively, our study demonstrated that BA could alleviate SU-induced cardiotoxicity by decreasing SU-increased cardiomyocyte injury, apoptosis, and fibrosis. Our study may provide potential therapeutic strategies for SU-induced cardiotoxicity.

## Data Availability

The original contributions presented in the study are included in the article/supplementary material, further inquiries can be directed to the corresponding authors.
